# The Burden of Disease Attributable to Diet Low in Vegetables From 1990 to 2021: A Global Analysis

**DOI:** 10.1002/fsn3.72298

**Published:** 2026-09-15

**Authors:** Xindi Wei, Xiaomeng Zhang, Xiaolei Lv, Hongchang Lai, Zhuoli Huang

**Affiliations:** ^1^ Department of Oral and Maxillofacial Implantology, Shanghai Ninth People's Hospital Shanghai Jiao Tong University School of Medicine Shanghai China; ^2^ College of Stomatology Shanghai Jiao Tong University Shanghai China; ^3^ National Center for Stomatology Shanghai China; ^4^ National Clinical Research Center for Oral Diseases Shanghai China; ^5^ Shanghai Key Laboratory of Stomatology Shanghai China; ^6^ Shanghai Research Institute of Stomatology Shanghai China

**Keywords:** disability‐adjusted life years, Global Burden of Disease Study, mortality, summary exposure value, vegetable

## Abstract

The global epidemiological landscape of diseases attributable to diet low in vegetables remains insufficiently characterized. This study aims to provide a comprehensive assessment of the disease burden associated with this risk on a global scale. Using data from the Global Burden of Diseases, Injuries, and Risk Factors Study 2021, the summary exposure value (SEV) of diet low in vegetables and the associated burden from 1990 to 2021 were analyzed. Trends of deaths and disability‐adjusted life years (DALYs) attributed to diet low in vegetables were measured using the age‐standardized rate (ASR) and the estimated annual percentage change (EAPC). The relationship between health outcomes and the Socio‐demographic Index (SDI) was explored to identify structural inequalities. Globally, the SEV attributable to diet low in vegetables was 27.34 (95% UI: 17.17–32.91) in 2021. Between 1990 and 2021, the SEV showed a continuous decline, with an EAPC of −0.72 (95% CI: −0.84 to −0.60). Central Sub‐Saharan Africa had the highest SEVs of diet low in vegetables, while East Asia had the lowest. The rates of deaths and DALYs attributed to diet low in vegetables have consistently decreased between 1990 and 2021. The EAPC for age‐standardized mortality rate (ASMR) was −2.01 (95% CI: −2.12 to −1.88), and the EAPC for age‐standardized DALY rate (ASDR) was −2.06 (95% CI: −2.21 to −1.91). For both deaths and DALYs, males consistently had higher numbers and rates than females. Furthermore, regions with lower SDIs faced a greater burden compared to higher SDI counterparts. Globally, cardiovascular diseases constituted the primary component of the burden associated with this dietary risk, followed by diabetes and kidney diseases. Although the global burden attributable to a diet low in vegetables has decreased, intake levels continue to vary across regions. These findings provide a descriptive foundation for identifying high‐risk populations.

## Graphical abstract

Using data from the Global Burden of Disease (GBD) 2021, temporal trends and disparities in disease burden attributable to diet low in vegetables from 1990 to 2021 were assessed. Globally, summary exposure value (SEV) and the age‐standardized rates of deaths and disability‐adjusted life years (DALYs) attributable to diet low in vegetables declined over the study period, whereas the absolute numbers of deaths and DALYs increased. A greater age‐standardized disease burden was observed in lower‐SDI regions. Notably, cardiovascular diseases accounted for the largest attributable burden. (Created with BioRender.com).

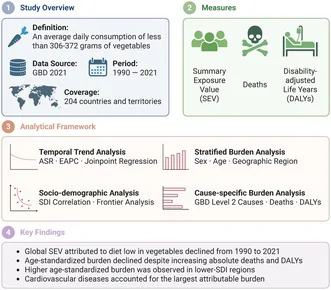

## 1 Background

Vegetables are widely promoted as healthy and have traditionally been a key component of dietary recommendations due to high concentrations of vitamins, minerals, dietary fibers, and phytochemicals. The compounds of vegetables have the potential to prevent or reduce the oxidation of DNA and impact cellular signal transduction pathways mediating cell proliferation and apoptosis (Liu 2013). Diets rich in vegetables are widely recommended across various countries for their beneficial effects on health. For instance, the Dietary Guidelines for Americans 2010 advise individuals to allocate half of their plate to fruits and vegetables (Slavin and Lloyd 2012). Numerous epidemiological studies have shown that higher vegetable intake is associated with lower risks of cardiovascular disease, several cancers, frailty, depression, and all‐cause mortality, highlighting vegetables as a key component of a healthy diet (Azupogo et al. 2018; Gao et al. 2019; Marmot 2011; Mo et al. 2019; Nam et al. 2022; Saglimbene et al. 2019; Sahashi et al. 2022; Su et al. 2023; Thomson and Thompson 2013; Wu et al. 2018; Yuan et al. 2020; Zhang et al. 2024).

The Global Burden of Diseases, Injuries, and Risk Factors Study (GBD) 2021 systematically gathered and consolidated the dietary diet data for 15 foods and nutrients from multiple resources (G. B. D. C. o. D. Collaborators 2024), providing the foundation for the secondary ecological analysis of modeled estimates derived from the GBD comparative risk assessment framework. Existing studies have mainly investigated the health burden associated with fruit and vegetable intake or examined specific outcomes such as cardiovascular diseases attributable to diet low in vegetables (He et al. 2025; Mao and Kong 2024; Xu et al. 2025). However, a comprehensive assessment focusing exclusively on diet low in vegetables remains lacking. In particular, the long‐term trends in exposure, cause‐specific disease burden across all GBD Level 2 causes, socioeconomic inequalities, and the global heterogeneity attributable to low vegetable intake have not been systematically characterized.

To address these gaps, this study used estimates from the GBD 2021 to systematically assess the summary exposure value (SEV), deaths, and disability‐adjusted life years (DALYs) attributable to diet low in vegetables from 1990 to 2021. To establish a descriptive foundation, the spatiotemporal trends in the SEV and the estimated burden attributed to diet low in vegetables were initially analyzed at global, regional, and national levels. Building upon this baseline, the heterogeneity of this burden was further explored by evaluating its relationship with socio‐demographic development alongside demographic factors such as sex and age. To deepen this exploration, the specific mortality and DALYs attributed to diet low in vegetables were subsequently delineated to characterize the underlying disease patterns. Examining these modeled trends offers descriptive insights that can inform future research and provide context for policy considerations across regions with different levels of socio‐demographic development.

## 2 Methods

### 2.1 GBD Source

The 2021 GBD study offers a comprehensive assessment of the health impacts of 369 diseases, injuries, and impairments, alongside 88 risk factors, across 204 countries and territories (G. B. D. C. o. D. Collaborators 2024; G. B. D. R. F. Collaborators 2020). SEVs, a relative risk‐weighted construct, were downloaded from the Global Health Data Exchange query tool (http://ghdx.healthdata.org/gbd‐results‐tool) (Hu et al. 2023). The SEV compares the distribution of excess risk times exposure level to a population where everyone is at maximum risk. The maximum risk in the denominator of the SEV is established by the relative risk corresponding to the 99th percentile of the worldwide exposure distribution. SEV values are assessed on a scale from 0 to 100, where 100 represents the situation where the entire population faces the maximum risk, and 0 signifies the situation where the entire population experiences minimum risk (G. B. D. R. F. Collaborators 2020). Age‐standardized and annual deaths and DALYs for numbers of a diet low in vegetables from 1990 to 2021 by region, sex, and country were obtained from the Global Health Data Exchange query tool as well. Age‐standardized rates (ASR) and estimated annual percentage change (EAPC) were employed to analyze the SEV trends of diet low in vegetables and the mortality and DALYs trends of diseases attributed to diet low in vegetables. ASR trends were utilized to depict changes in disease patterns more accurately within populations, while EAPC summaries quantified ASR trends across different populations over specific time frames. Socio‐demographic Index (SDI) scores, reflecting the socio‐economic and developmental statuses of countries and regions, were used to categorize the 204 entities into five regions: low‐SDI, low‐middle‐SDI, middle‐SDI, high‐middle‐SDI, and high‐SDI quintiles. Higher SDI values corresponded to increased socio‐economic and developmental levels within regions, aiding policymakers in comprehending the overall development and health statuses of diverse areas (Wu et al. 2024). Additionally, the world was segmented into 21 geographic regions. This research adhered to the Guidelines for Accurate and Transparent Health Estimates Reporting (GATHER) (Stevens et al. 2016).

### 2.2 Estimates

The GBD 2021 Collaborators modeled deaths and DALYs estimates attributed to diet low in vegetables for male and female participants across the following age groups: 25–29 years, 30–34 years, 35–39 years, 40–44 years, 45–49 years, 50–54 years, 55–59 years, 60–64 years, 65–69 years, 70–74 years, 75–79 years, 80–84 years, 85–89 years, 90–94 years and 95+ years. The Cause of Death Ensemble model (CODEm) was used to estimate the death rates, and the Bayesian meta‐regression tool DisMod‐MR 2.1 software was developed to conduct estimations through an analytical cascade process (G. B. D. C. o. D. Collaborators 2024).

### 2.3 Definition

The definition of a diet low in vegetables was characterized by an average daily consumption of less than 306–372 g of vegetables (including fresh, frozen, cooked, canned, or dried vegetables and excluding legumes and salted or pickled vegetables, juices, nuts and seeds, and starchy vegetables such as potatoes or corn) (G. B. D. R. F. Collaborators 2020). This range represents the level of exposure associated with the lowest disease risk. Within the GBD framework, the same definition is applied consistently across age groups, sexes, and geographic regions. All estimates of attributable deaths and DALYs in the present study were derived directly from the standardized GBD comparative risk assessment methodology using this predefined exposure definition.

### 2.4 Joinpoint Analysis

Temporal trends in ASRs were evaluated using the Joinpoint Regression Program (version 6.1.0, National Cancer Institute, Bethesda, MD, USA). Separate analyses were performed for males, females, and both sexes combined. ASRs were specified as the dependent variable, and calendar year (1990–2021) was treated as the independent variable. The Grid Search algorithm was used to identify candidate joinpoints, with the minimum and maximum numbers of joinpoints set to 0 and 5, respectively. The optimal model was selected using the Monte Carlo permutation test with an overall significance level of 0.05 based on 4499 permutations. Annual percent change (APC) and average annual percent change (AAPC) with 95% confidence intervals (CIs) were estimated using the parametric method. The AAPC was calculated over the entire study period (1990–2021) to summarize the overall temporal trend. An increasing trend was inferred when both the AAPC estimate and its 95% CI upper and lower limits exceed 0. Conversely, a decreasing trend was indicated when both the AAPC estimate and its upper and lower 95% CI limits are below 0.

### 2.5 Frontier Analysis

Frontier analysis was performed to evaluate the relationship between the age‐standardized DALY rate attributable to diet low in vegetables and the SDI. The frontier represents the lowest empirically observed age‐standardized DALY rate achieved at a given level of SDI and is used as a benchmark for comparing disease burden across countries and regions.

A non‐parametric bootstrap algorithm implemented in R was used to estimate the frontier. Age‐standardized DALY rates (ASDR) were matched with the corresponding SDI values for each country‐year observation. To improve the robustness of the frontier estimation, 100 bootstrap samples were generated by randomly resampling the complete set of country‐year observations with replacement. Within each bootstrap sample, observations were ordered according to increasing SDI and, for observations with similar SDI values, by decreasing ASDR. Each observation was sequentially excluded to identify super‐efficient points, defined as observations lying below the frontier estimated from the remaining observations. These super‐efficient observations were excluded before reconstructing the frontier. The frontier was then estimated as the cumulative minimum ASDR across increasing SDI values, resulting in a monotonic non‐increasing lower boundary of the observed burden. Frontier estimates from all bootstrap samples were averaged to obtain the final frontier estimate for each country‐year observation.

The effective difference was calculated as the observed ASDR minus the corresponding frontier estimate, representing the gap between the observed burden and the empirically achievable burden at a comparable level of socio‐demographic development. Larger effective differences indicate greater potential for burden reduction. For visualization, the bootstrap‐averaged frontier estimates were smoothed against SDI using locally estimated scatterplot smoothing (LOESS) with a span parameter of 0.2.

### 2.6 Statistical Analysis

Within the GBD framework, 95% uncertainty intervals (UIs) were estimated from 1000 uncertainty draws, with the lower and upper bounds defined as the 2.5th and 97.5th percentiles of the draws (G. B. D. R. F. Collaborators 2020). The 95% UIs used in this study were obtained directly from the GBD 2021 estimates. Secondary analyses were performed using the point estimates of the ASRs rather than the full set of posterior draws. For EAPC calculation, a log‐linear regression model was fitted as $\ln\left(\mathrm{ASR}\right)=\alpha +\beta \times \text{calendar year}+\varepsilon$, where ASR represents the age‐standardized rate and β is the regression coefficient for calendar year. The EAPC was calculated as $100\times \left[\exp\left(\beta \right)-1\right]$, and its 95% confidence interval was estimated as $100\times \left[\exp\left(\beta \pm 1.96\times SE_{\beta }\right)-1\right]$. If both the EAPC value and the upper bound of the 95% CI were negative, the ASR was deemed to have a decreasing trend. Conversely, if both the EAPC value and the lower bound of the 95% CI were positive, the ASR was considered to have an increasing trend. If the 95% CI centered on 0, the ASR was regarded as stable. All statistical analyses were conducted using R software (version 4.3.1).

## 3 Results

### 3.1 Risk‐Weighted Exposure Attributed to a Diet Low in Vegetables

The magnitude of exposure attributed to a diet low in vegetables was evaluated using the age‐standardized SEV. In 2021, the global SEVs of low vegetable diets were 27.34 (95% UI 17.17–32.91). In general, Central Sub‐Saharan Africa had the highest SEVs attributed to diet of low vegetables, whereas East Asia had the lowest SEVs attributed to diet low in vegetables. At the global level, the SEVs attributed to diet low in vegetables have decreased in the past 30 years (EAPC = −0.72, 95% CI −0.94 to −0.60). At the regional level, most regions showed a decrease in SEVs for diet low in vegetables. The most pronounced decrease in ASR was in East Asia (EAPC = −7.04, 95% CI −7.33 to −6.75) and high‐middle SDI region (EAPC = −2.22, 95% CI −2.48 to −1.95). It should be noted that Oceania, Eastern Europe, Australasia, Western Europe, and High‐income North America showed increasing trends of SEVs attributed to diet low in vegetables during 1990 to 2021 (Table 1, Table S1, Figure S1).

**TABLE 1:** The age‐standardized SEV and temporal trends attributed to diet low in vegetables in 1990 and 2021.

Location	1990	2021	1990–2021
	ASR per 100,000 population (95% UI)	ASR per 100,000 population (95% UI)	EAPC (95% CI)
Global	33.90 (20.60, 40.59)	27.34 (17.17, 32.91)	−0.72 (−0.84, −0.60)
High SDI	25.13 (16.58, 32.04)	22.76 (14.53, 29.27)	−0.17 (−0.24, −0.10)
High‐middle SDI	21.46 (13.99,27.85)	11.21 (7.36, 15.19)	−2.22 (−2.48, −1.95)
Low SDI	57.31 (30.29, 63.48)	54.34 (29.81,60.96)	−0.18 (−0.2, −0.16)
Low‐middle SDI	47.43 (26.71, 54.12)	40.21 (24.25, 47.23)	−0.51 (−0.56, −0.47)
Middle SDI	37.08 (22.30, 43.83)	23.7 (15.66, 29.00)	−1.6 (−1.77, −1.43)

Abbreviations: ASR, age standardized incidence rate; CI, confidence interval; EAPC, estimated annual percentage change; UI, uncertainty interval.

### 3.2 Disease Burden Attributed to Diet Low in Vegetables in 2021

In 2021, the global deaths attributed to diet low in vegetables remained substantial, with a total of 861,480 cases (95% UI 541,278–1,189,503) and an age‐standardized mortality rate (ASMR) of 10.35 per 100,000 persons (95% UI 6.56–14.30). The global DALYs for diseases attributed to diet low in vegetables were 20,655,691 (95% UI 12,448,914–28,673,802), with an age‐standardized DALY rate (ASDR) of 241.70 per 100,000 persons (95% UI 146.31–335.38). The regions with the highest ASMR and ASDR for both sexes combined in 2021 were the low SDI quintile and Central Sub‐Saharan Africa (Tables 2 and 3, Tables S2 and S3).

**TABLE 2:** The deaths cases, age‐standardized deaths, and temporal trends attributed to diet low in vegetables in 1990 and 2021.

Location	1990		2021		1990–2021
	Deaths cases no. (95% UI)	ASR per 100,000 population (95% UI)	Deaths cases no. (95% UI)	ASR per 100,000 population (95% UI)	EAPC (95% CI)
Global	686,077 (395,488, 963,703)	18.96 (11.06, 26.47)	861,480 (541,278, 1,189,503)	10.35 (6.56, 14.30)	−2.01 (−2.13, −1.88)
High SDI	96,899 (51,965, 146,121)	8.88 (4.79, 13.38)	130,517 (84,535, 181,603)	5.55 (3.5, 7.76)	−1.24 (−1.37, −1.11)
High‐middle SDI	118,560 (62,945, 175,599)	13.51 (7.30, 19.78)	92,732 (56,604, 136,988)	4.91 (3.04, 7.25)	−3.34 (−3.42, −3.26)
Low SDI	88,628 (48,637, 123,566)	45.54 (27.15, 62.25)	147,686 (83,885, 204,090)	34.34 (20.77, 47.01)	−1.04 (−1.14, −0.93)
Low‐middle SDI	147,756 (82,559, 207,184)	27.66 (16.30, 38.44)	245,660 (152,207, 338,384)	19.33 (12.14, 26.39)	−1.20 (−1.28, −1.11)
Middle SDI	233,469 (140,765, 322,408)	27.56 (17.32, 37.26)	243,879 (154,910, 338,738)	10.18 (6.55, 14.07)	−3.37 (−3.57, −3.17)

Abbreviations: ASR, age standardized incidence rate; CI, confidence interval; EAPC, estimated annual percentage change; UI, uncertainty interval.

**TABLE 3:** The DALYs cases, age‐standardized DALYs, and temporal trends attributed to diet low in vegetables in 1990 and 2021.

Location	1990		2021		1990–2021
	DALYs cases no. (95% UI)	ASR per 100,000 population (95% UI)	DALYs cases no. (95% UI)	ASR per 100,000 population (95% UI)	EAPC (95% CI)
Global	17,756,189 (9,735,693, 25,084,219)	442.84 (248.95, 625.16)	20,655,691 (12,448,914, 28,673,802)	241.70 (146.31, 335.38)	−2.06 (−2.21, −1.91)
High SDI	2,028,178 (1,085,584, 3,059,637)	187.78 (100.46, 283.64)	2,404,711 (1,533,007, 3,375,028)	120.99 (77.33, 170.63)	−1.10 (−1.25, −0.95)
High‐middle SDI	2,825,121 (1,443,940, 4,214,267)	291.59 (152.17, 432.05)	1,718,102 (1,018,274, 2,574,564)	90.15 (53.84, 134.43)	−4.01 (−4.14, −3.88)
Low SDI	2,528,276 (1,294,387, 3,569,801)	1075.28 (579.17, 1502.02)	4,182,999 (2,224,672, 5,920,653)	783.95 (435.41, 1093.78)	−1.21 (−1.30, −1.11)
Low‐middle SDI	4,233,658 (2,215,869, 5,921,007)	660.49 (358.88, 924.49)	6,456,176 (3,824,899, 8,970,538)	440.97 (266.9, 612.1)	−1.36 (−1.43, −1.28)
Middle SDI	6,121,681 (3,558,203, 8,535,416)	594.84 (357.15, 821.03)	5,870,045 (3,693,433, 8,170,388)	222.05 (140.62, 309.52)	−3.38 (−3.62, −3.14)

Abbreviations: ASR, age standardized incidence rate; CI, confidence interval; EAPC, estimated annual percentage change; UI, uncertainty interval.

**FIGURE 1:**
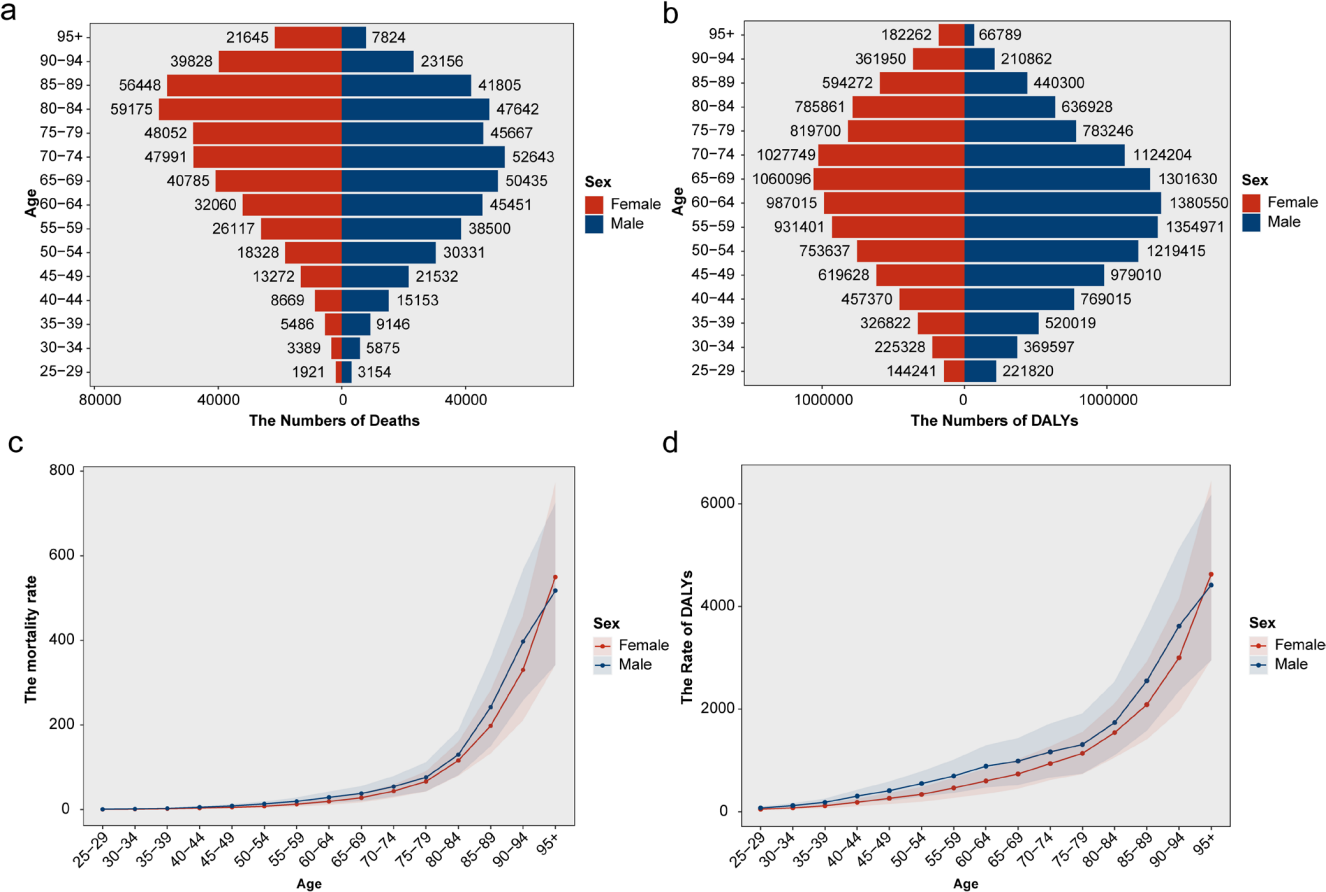
Age‐specific mortality as well as DALY numbers and rates of diseases attributed to diet low in vegetables in 2021. Age‐specific (a) death number and (b) DALY number of males and females. Trends of (c) mortality rate and (d) DALY rate for different age groups. Shaded regions represent the 95% UI.

Among males, the number of DALYs peaked in the 60–64‐year age group and the number of deaths peaked in the 70–74‐year age group, whereas among females, the corresponding peaks occurred in the 65–69‐year and 80–84‐year age groups, respectively. After the age of 75, the number of deaths attributed to diet low in vegetables among females was higher than that among males, while before the age of 74, the number among males was higher than that among females. Both the numbers of deaths and DALYs for males and females demonstrated a pattern of lower values at the extremes and higher values in the center (Figure 1a,b). The mortality and DALY rates exhibited a consistent upward trajectory until the age of 80, with males consistently exhibiting slightly higher rates than females. After the age of 80, these rates start to rise sharply. Both the mortality and DALY rates peaked at 95+ years for males and females (Figure 1c,d).

### 3.3 The Trend of Disease Burden Attributed to Diet Low in Vegetables From 1990 to 2021

**FIGURE 2:**
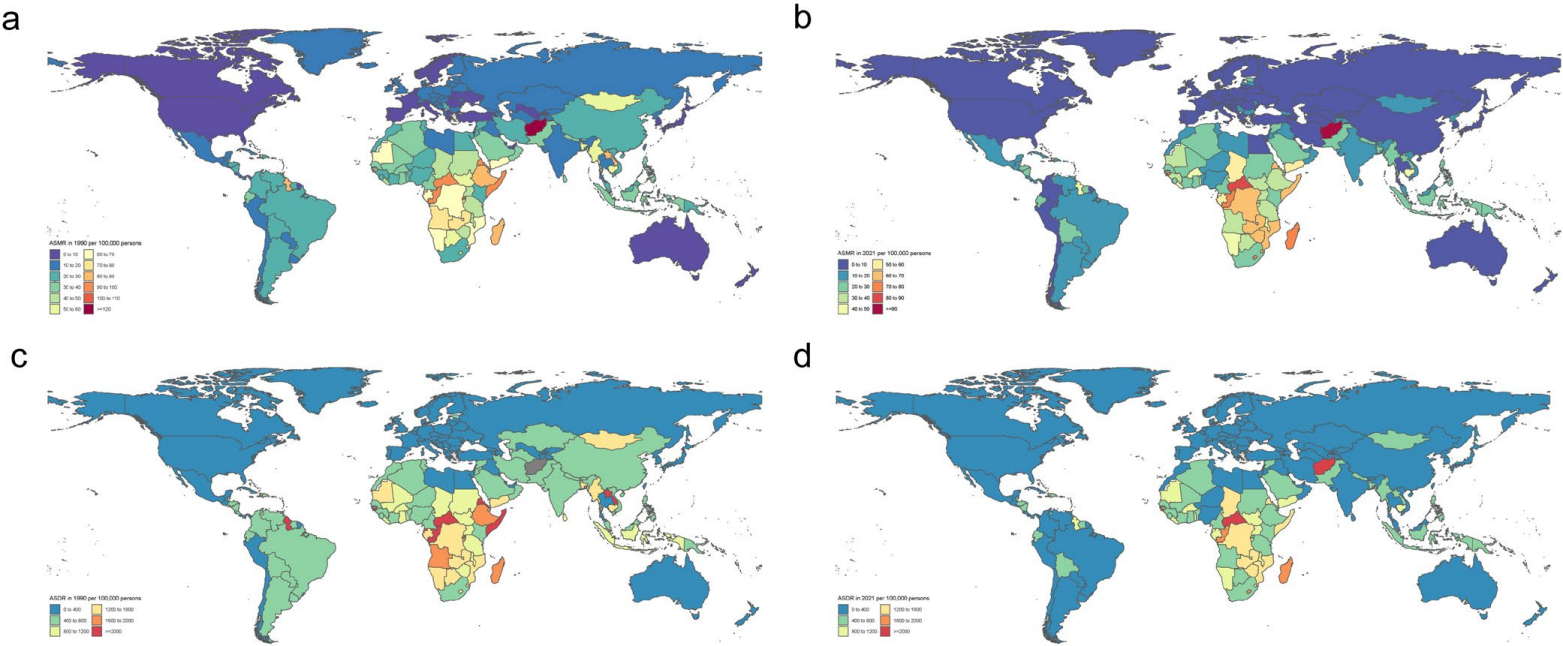
The ASR of diet low in vegetables related deaths and DALYs in countries and territories in 1990 and 2021. The ASMR attributed to diet low in vegetables in (a) 1990, (b) 2021. The ASDR attributed to diet low in vegetables in (c) 1990, (d) 2021.

From 1990 to 2021, the death number of diseases attributed to diet low in vegetables increased from 686,077 (95% UI 395,488–963,703) to 861,480 (95% UI 541,278–1,189,503), marking 25.57% increase. DALYs increased from17,756,189 to 20,655,691, corresponding to a 16.33% growth over the period. Despite the significant rise in the absolute number of death cases, the ASMR demonstrated a decrease from 18.96 per 100,000 persons (95% UI 11.06–26.47) in 1990 to10.35 per 100,000 persons (95% UI 6.56–14.30) in 2021. The EAPC for ASMR was −2.01 (95% CI −2.13 to −1.88). The most pronounced decrease in ASR of mortality was in East Asia (EAPC = −7.69, 95% CI −7.93 to −7.44) and high‐middle SDI region (EAPC = −3.34, 95% CI −3.42 to −3.26). In addition, the ASDR demonstrated decrease from 442.84 per 100,000 persons (95% UI 248.95–625.16) in 1990 to 241.70 per 100,000 persons (95% UI 146.31–335.38) in 2021. The EAPC for ASDR was −2.06 (95% CI −2.21 to −1.91). The most pronounced decrease in ASR of DALY was in East Asia (EAPC = −8.66, 95% CI −8.99 to −8.32) as well as high‐middle SDI region (EAPC = −4.01, 95% CI −4.14 to −3.88) (Tables 2 and 3, Figure 2, Tables S2 and S3).

Globally, the ASMR decreased most significantly during 1995 to 1998 with an APC of −3.015 (95% CI −3.209 to −2.820) in male and −3.170 (95% CI −4.037 to −2.295) in female, respectively (Figure S2). During 1990 to 2019, the ASMR significantly decreased with an AAPC of −2.046 (95% CI −2.117 to −1.974) in male and −1.818 (95% CI −1.928 to −1.708) in female. In both sexes combined, the ASMR significantly decreased with an AAPC of −1.928 (95% CI −2.037 to −1.819) (Table 4).

**TABLE 4:** Trends in ASMR among both sexes, males, and females, 1990–2021.

Gender	Period	APC (95% CI)	AAPC (95% CI)
Both	1990–1995	−2.147 (−2.342, −1.952)	−1.928 (−2.037, −1.819)
	1995–1998	−3.184 (−4.045, −2.315)	
	1998–2007	−2.454 (−2.547, −2.360)	
	2007–2012	−1.713 (−1.975, −1.450)	
	2012–2016	−0.708 (−1.106, −0.309)	
	2016–2021	−1.182 (−1.360, −1.004)	
Male	1990–1995	−1.967 (−2.159, −1.774)	−2.046 (−2.117, −1.974)
	1995–1998	−3.015 (−3.209, −2.820)	
	1998–2007	−2.343 (−2.655, −2.030)	
	2007–2012	−2.839 (−3.147, −2.531)	
	2012–2016	−1.879 (−2.071, −1.687)	
	2016–2021	−1.142 (−1.195, −1.088)	
Female	1990–1995	−2.212 (−2.407, −2.017)	−1.818 (−1.928, −1.708)
	1995–1998	−3.170 (−4.037, −2.295)	
	1998–2007	−2.325 (−2.419, −2.230)	
	2007–2012	−1.577 (−1.989, −1.164)	
	2012–2016	−0.522 (−0.777, −0.267)	
	2016–2021	−1.170 (−1.349, −0.991)	

Abbreviations: AAPC, average annual percent change; APC, annual percent change.

**FIGURE 3:**
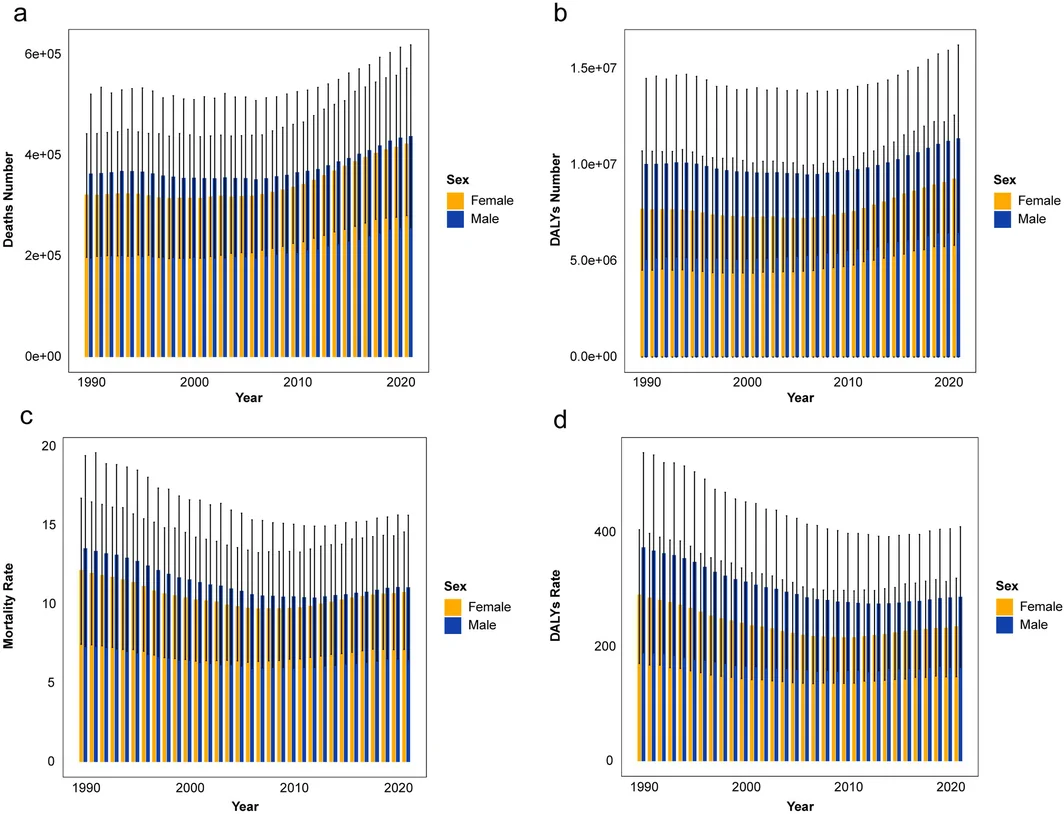
Trends in numbers and rates attributed to diet low in vegetables related mortality and DALY by sex from 1990 to 2021. Trends in (a) mortality number, (b) DALY number, (c) mortality rate, and (d) DALY rate from 1990 to 2021.

Figure 3 depicted the trends in the sex‐specific all‐age numbers and rates of mortality and DALY attributed to diet low in vegetables from 1990 to 2021. For both males and females, both the mortality and DALY numbers were generally increasing (Figure 3a,b), while the rates were decreasing, with males consistently exhibiting slightly higher numbers and rates than females (Figure 3c,d).

Age‐specific temporal patterns of deaths and DALY rates attributable to diet low in vegetables are presented in Figure S3. Overall, death and DALY rates declined over time across most age groups, whereas the burden increased markedly with age, with the highest rates consistently observed among individuals aged 85 years and older.

### 3.4 The Association Between SDI and Disease Burden Attributed to Diet Low in Vegetables

**FIGURE 4:**
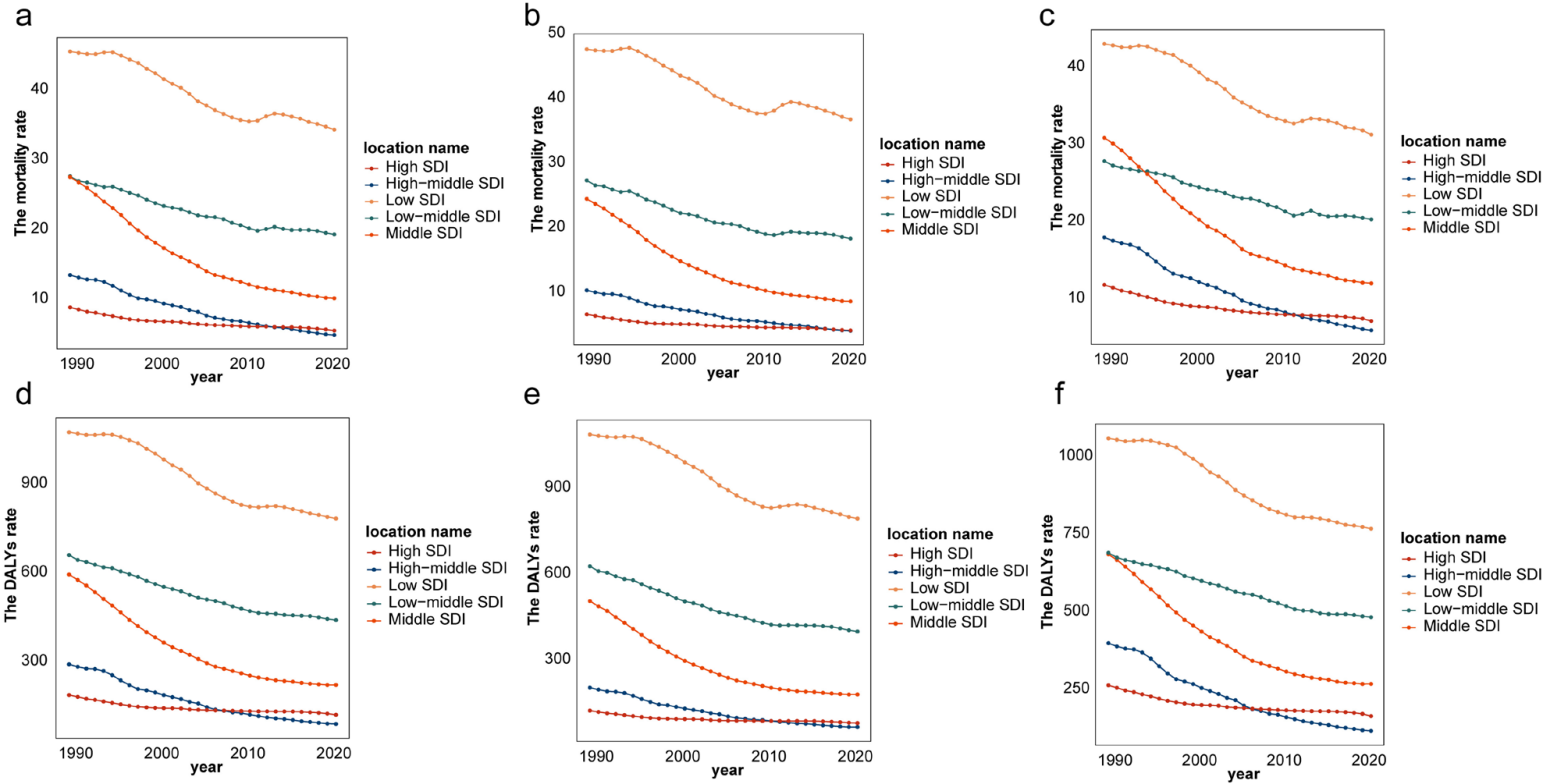
The ASR of diet low in vegetables related deaths and DALYs in both sexes combined, females, and males among different SDI regions. The ASR of diet low in vegetables related deaths among different regions (a) In both sexes combined, (b) Females, and (c) Males. The ASR of diet low in vegetables related DALYs among different regions (d) In both sexes combined, (e) Females, and (f) Males.

**FIGURE 5:**
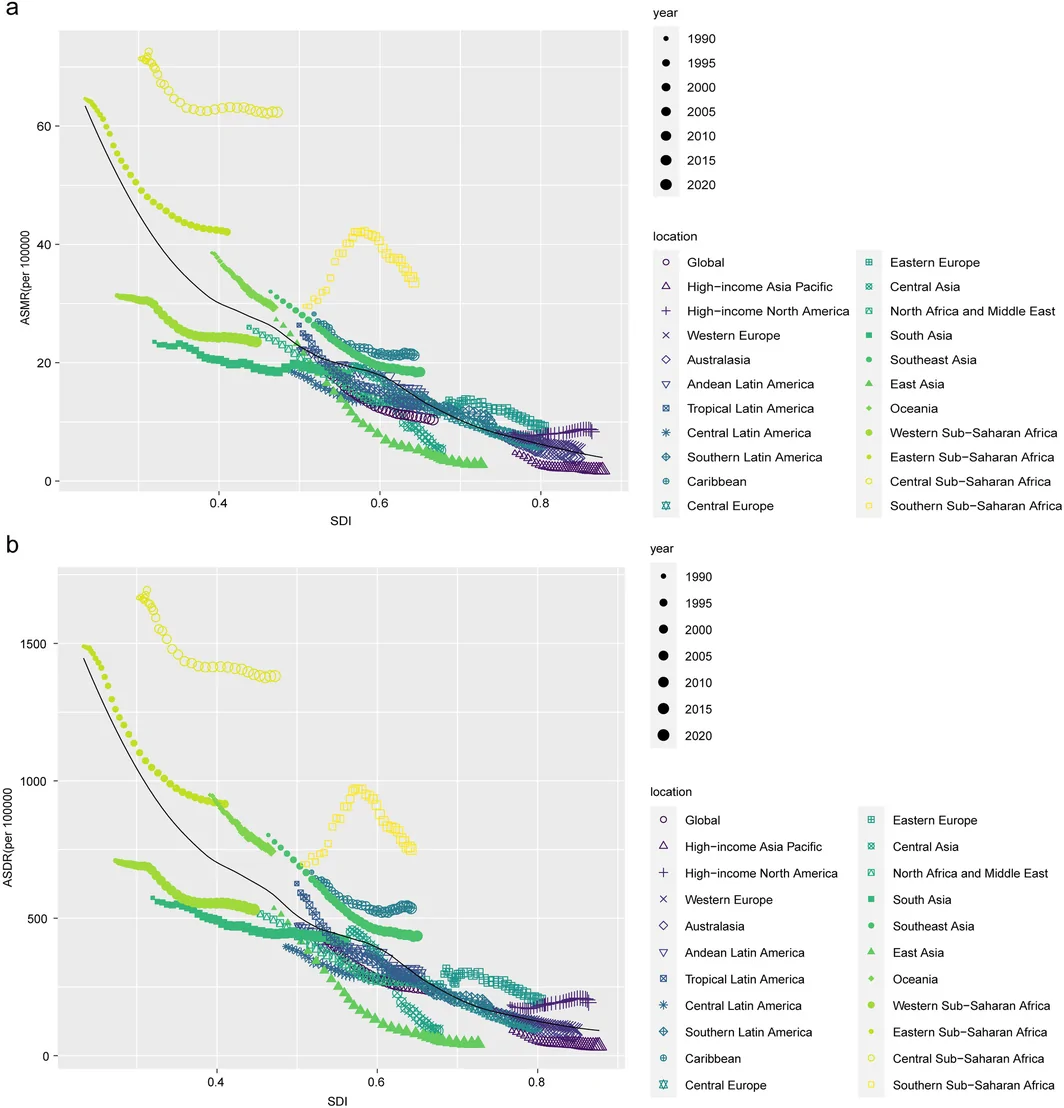
Correlation of disease burden attributed to diet low in vegetables and SDI at regional level from 1990 to 2021 (a) ASMR attributed to diet low in vegetables by SDI from 1990 to 2021 (b) ASDR attributed to diet low in vegetables by SDI from 1990 to 2021. Black line represented expected values of ASMR and ASDR on the basis of SDIs in all regions.

**FIGURE 6:**
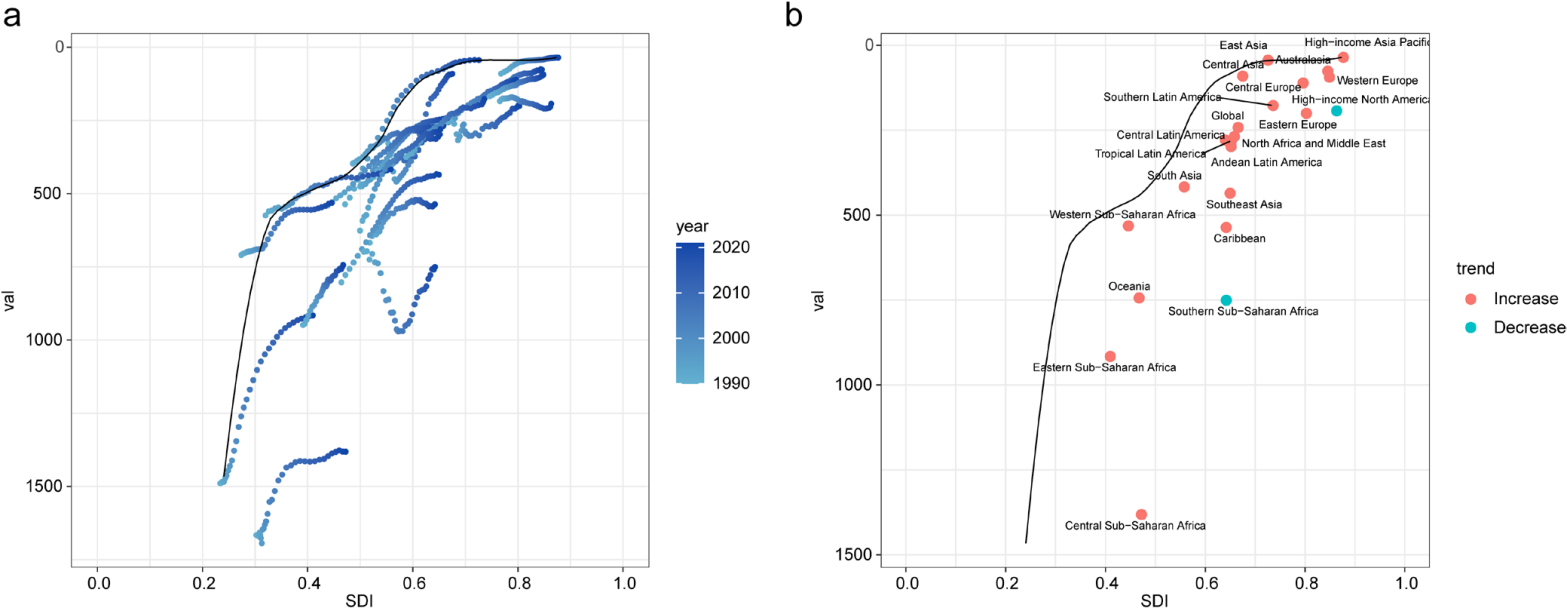
Frontier analysis based on SDI and ASDR in 2021. (a) The unrealized health gains of countries or regions at different levels of development during the period 1990–2021. (b) The DALYs burden and the effective difference in regions with different SDI levels in 2021. The frontier was delineated in solid black color, and regions were represented as dots. Red dots indicated a decrease in ASDR from 1990 to 2021, while blue dots indicated an increase in ASDR during 1990 to 2021.

To understand the profound geographic heterogeneity observed in the disease burden, the role of SDI was evaluated. Since 1990, the ASR of diet low in vegetables related deaths and DALYs decreased for males and females in all SDI regions. In both sexes combined, males and females, lower SDI regions had higher deaths and DALYs rates attributed to diet low in vegetables (Figure 4). At the regional level, SDI was negatively correlated with both the ASMR and ASDR attributable to diet low in vegetables (Figure 5). Spearman rank correlation analysis demonstrated strong inverse associations between SDI and ASMR (*ρ* = −0.86, *p* < 0.05) and between SDI and ASDR (*ρ* = −0.86, *p* < 0.05). A similar inverse relationship was visually observed at the national level (Figure S4). The Central Sub − Saharan Africa had lower SDI as well as higher ASMR and ASDR attributed to diet low in vegetables. In contrast, the high‐income Asia Pacific had lower ASMR and ASDR. Frontier analysis was utilized to identify regions where the observed burden differed from that of comparable regions at similar levels of development. The unrealized health gains of regions at different levels of development during the period 1990–2021 were shown in Figure 6a. With socio‐demographic development, the effective difference generally decreased, highlighting that low‐ and low‐middle SDI regions possess the greatest unrealized health gains (Figure 6b).

### 3.5 Disease‐Specific Burden Attributable to Diet Low in Vegetables

**FIGURE 7:**
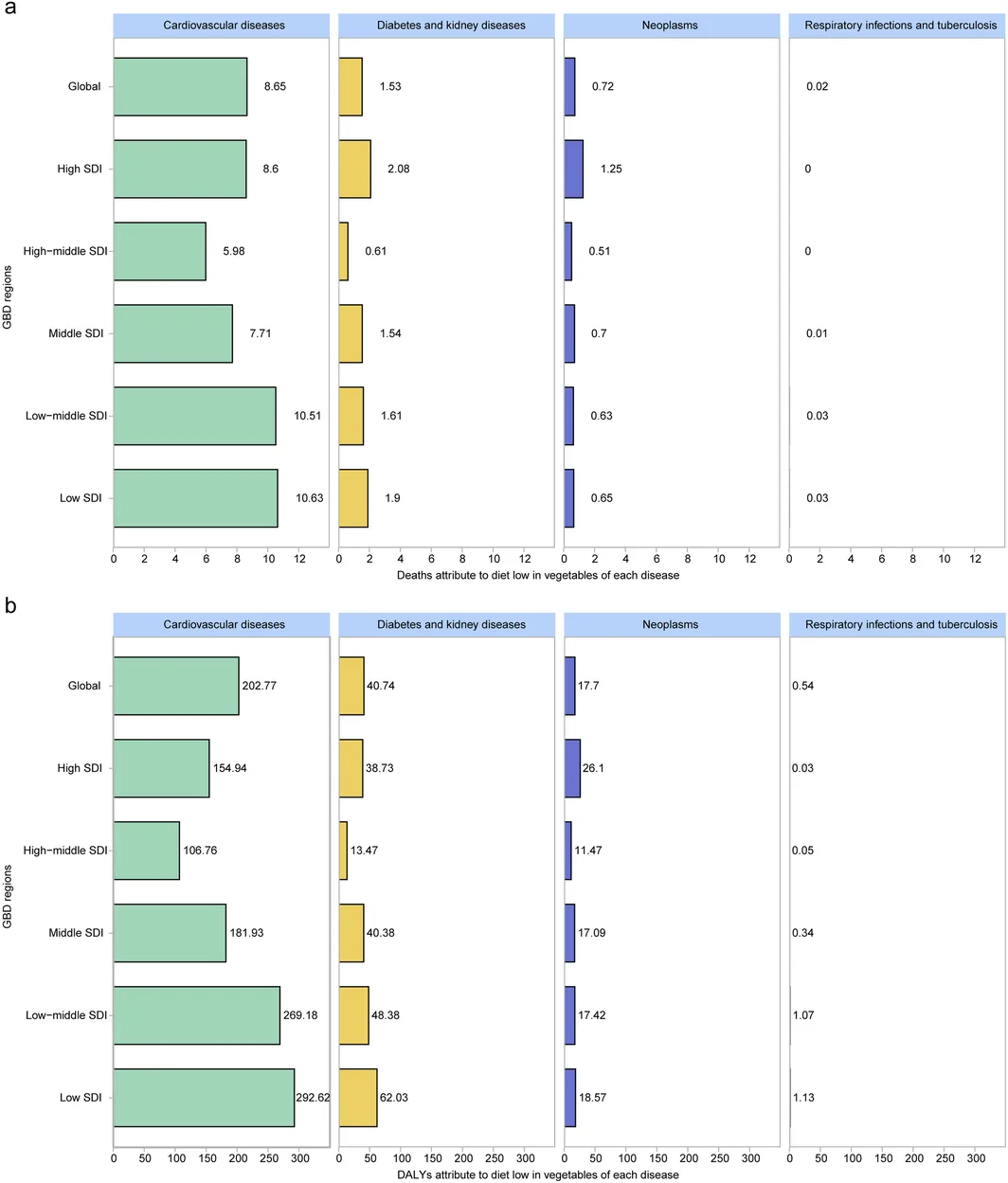
Disease burden attributable to a diet low in vegetables on GBD level 2 cause (a) Deaths attributable to diet low in vegetables (b) DALYs attributable to diet low in vegetables.

Globally, at GBD Level 2, cardiovascular diseases accounted for the largest proportion of deaths attributable to this dietary risk, followed by diabetes and kidney diseases. In 2021, the mortality rate for cardiovascular diseases associated with a diet low in vegetables peaked at 8.65, while rates for diabetes and kidney diseases were recorded at 1.53. The region with the highest mortality rate for cardiovascular diseases (rate = 10.63) and respiratory infections and tuberculosis (rate = 0.03) attributed to diet low in vegetables was the low SDI region. Conversely, for diabetes and kidney diseases (rate = 2.08), as well as neoplasms (rate = 1.25), the high SDI region exhibited the highest mortality rates (Figure 7a).

At GBD Level 2, globally, cardiovascular diseases accounted for the largest proportion of DALYs attributable to a diet low in vegetables, followed by diabetes and kidney diseases. In 2021, the DALY rate for cardiovascular diseases attributable to a diet low in vegetables stood at 202.77, while rates for diabetes and kidney diseases were at 40.74. The region with the highest DALY rate for cardiovascular diseases (rate = 292.62), diabetes and kidney diseases (rate = 62.03), and respiratory infections and tuberculosis (rate = 1.13) attributable to diet low in vegetables was the low SDI region. However, for neoplasms (rate = 26.1), the high SDI region displayed the highest DALY rate (Figure 7b).

## 4 Discussion

Based on GBD 2021, the SEV and temporal trends in deaths and DALYs attributed to diet low in vegetables at global, regional, and national levels were explored in this study. Large variations existed in the SEV and attributable disease burden across countries and territories. Some regions and countries had increased SEV and attributable burden. The attributable burden was heavier in males, indicating the gender gap. SDI correlations indicated that the disease burden was unequally distributed. In addition, cardiovascular diseases showed the highest burden of deaths and DALYs in association with diet low in vegetables. These findings provide descriptive information on global patterns and may serve as a basis for generating hypotheses or guiding future research on dietary risk and public health priorities.

This study observed a divergence between the rising absolute burden and the declining ASRs. ASRs are adjusted for changes in population age structure and therefore primarily reflect changes in disease risk over time. In contrast, the absolute numbers of deaths and DALYs are strongly influenced by population size and age composition. Although age‐specific disease burden has declined, continued population growth has increased the total number of individuals at risk, while population aging has expanded the proportion of older adults (Kanasi et al. 2016).

Despite a decrease in the global prevalence of diets low in vegetables in the past 30 years, the issue remains severe in Central Sub‐Saharan Africa. In low‐SDI countries in Africa, populations may rely more heavily on staple foods such as rice or cassava, potentially limiting dietary diversity (Adamolekun and Hiffler 2017). Although the underlying causes are likely multifactorial, including constraints related to food availability and affordability, these findings suggest that improving dietary diversity may warrant consideration in future nutrition and agricultural policies (Slavin and Lloyd 2012).

The decline in age‐standardized mortality and DALY rates attributable to a diet low in vegetables may reflect multiple concurrent changes, including improvements in healthcare and developments in agricultural technology. Recent advances in vegetable production and postharvest processing, including vertical farming and technologies designed to better preserve the nutritional quality of fruits and vegetables, illustrate broader efforts to improve the availability and quality of plant foods (Shanmugam et al. 2025; Sowmya et al. 2024). However, significant differences in death and DALY attributable to a diet low in vegetables between different SDI quintiles were still observed. The low SDI quintile has higher death and DALY rates attributed to a diet low in vegetables than the high SDI quintile from 1990 to 2021. Dietary recommendations in regions with high SDI advocate for a high vegetable intake (Slavin and Lloyd 2012). In contrast, dietary recommendations may be overlooked in low SDI regions. Moreover, the present domestic production of vegetables in low SDI regions may be insufficient to support everyone achieving the recommended vegetable intake due to the backwardness in agricultural technology. While this simple comparison demonstrates the trend that lower‐development regions face higher burdens, the frontier analysis provides a more critical epidemiological insight, quantifying the unrealized health gains. The persistence of a large effective difference in certain low‐SDI regions indicates that economic constraints alone cannot fully explain their high disease burden, and they possess substantial improvement potential even within their current socio‐economic constraints. Consequently, these results translate a static socio‐economic description into a concrete measure of achievable health improvement, providing a reference for future health promotion efforts and agricultural planning.

While the overall AAPC describes a long‐term global reduction in the modeled ASMR, the Joinpoint regression analysis provides a more granular temporal resolution. Specifically, the most rapid declines in ASMR were observed between 1995 and 1998. However, this trajectory slowed between 2012 and 2016, particularly among females. This observed deceleration suggests that earlier positive shifts in global dietary patterns or healthcare access may have temporarily plateaued during this timeframe that warrants focused, primary epidemiological investigation to understand the underlying mechanisms.

In this study, cardiovascular diseases accounted for the largest proportion of deaths and DALYs attributable to diet low in vegetables. This finding is biologically plausible because adequate vegetable consumption may reduce cardiovascular risk through multiple complementary pathways. Previous studies have reported that higher consumption of vegetables, particularly green leafy vegetables and those rich in vitamin C, is associated with lower risks of coronary heart disease and cardiovascular mortality (Joshipura et al. 2001; Wang et al. 2014). In addition, vegetable‐derived nitrate has been recognized as an important bioactive compound that enhances nitric oxide bioavailability, thereby improving vascular function (Bondonno et al. 2018). Because these mechanisms influence the development and progression of cardiovascular diseases, insufficient vegetable intake may have a greater impact on cardiovascular disease than on many other disease categories. However, the relative contribution of neoplasms to disease burden was more pronounced in high‐SDI regions. This pattern may reflect the combined influence of longer life expectancy, population aging, and a greater proportion of individuals surviving to ages at which cancer incidence increases substantially (Global Burden of Disease Cancer et al. 2022; Wang et al. 2025). However, these explanations remain speculative because the ecological nature of the GBD data does not permit direct assessment of the underlying mechanisms.

The ranking of disease categories was broadly consistent across mortality and DALY patterns, with cardiovascular diseases contributing the largest burden, followed by diabetes and kidney diseases, neoplasms, and respiratory infections and tuberculosis. However, DALYs provided additional information on the overall health burden by incorporating both premature mortality and years lived with disability. As a result, disparities across SDI levels were more apparent in DALY estimates than in mortality estimates.

In summary, this study provides a comprehensive descriptive and ecological analysis of the health burden attributed to diet low in vegetables, stratified by age, sex, and socio‐demographic development. However, this study is subject to certain limitations that warrant consideration. Firstly, the precision of the estimates could be compromised by variations in the quality and accessibility of raw data across different countries and regions. Particularly in low‐income countries and those with smaller populations, the absence of dependable data may lead to inaccuracies in estimating disease burdens. Second, the GBD methodology is dependent on various assumptions and modeling approaches, potentially introducing uncertainties into the estimations. Third, a notable limitation inherent to the GBD framework is the treatment of diet low in vegetables as a single exposure construct. This approach does not reflect real‐world dietary complexity and inevitably introduces structural misclassification into the exposure assessment. Different types of vegetables, along with varying preparation methods and broader dietary patterns, likely possess different relationships with disease burden. Therefore, future epidemiological studies are required to specifically explore the roles of different types of vegetables and detailed dietary matrices on health outcomes. Finally, this study is constrained by the limitations of cross‐national ecological attribution analyses. The observed geographic disparities and temporal trends may be confounded by factors unmeasured in this model, including international differences in surveillance quality, diagnostic capacities, healthcare access, demographic structures, competing risks, and broader dietary pattern context. Consequently, the findings should be strictly interpreted as descriptive and hypothesis‐generating, serving as a foundation for future individual‐level longitudinal research.

## 5 Conclusion

Globally, although ASMR and ASDR have declined, the absolute number of deaths and DALYs attributed to diet low in vegetables remains substantial, with variation observed across countries and SDI regions. As a descriptive, ecological analysis, this study cannot establish causal relationships or determine the effectiveness of specific interventions. However, the observed geographic and demographic disparities help to highlight vulnerable populations. These results provide context to inform the design of future, individual‐level epidemiological research and to guide broader public health discussions aimed at improving global vegetable consumption.

## Author Contributions

**Xindi Wei:** conceptualization, methodology, software, formal analysis, visualization, writing – original draft, investigation. **Xiaomeng Zhang:** writing – original draft, methodology, funding acquisition. **Xiaolei Lv:** validation, visualization, writing – review and editing. **Hongchang Lai:** writing – review and editing, supervision, funding acquisition. **Zhuoli Huang:** supervision, writing – review and editing.

## Funding

This study was supported by the Project of Healthcare Youth Talent from Shanghai Municipal Health Commission (2022YQ041), and the Biobank Project of Ninth People's Hospital affiliated Shanghai Jiao Tong University School of Medicine (YBKA202207).

## Disclosure

AI use disclosure: No generative AI tools were used in this study.

## Ethics Statement

The authors have nothing to report.

## Consent

The authors have nothing to report.

## Conflicts of Interest

The authors declare no conflicts of interest.

## Supporting information


**Figure S1:** The ASR of diet low in vegetables related SEV in countries and territories in 1990 and 2021. The ASR of diet low in vegetables related SEV in (a) 1990, (b) 2021.
**Figure S2:** Joinpoint regression analyses of the sex‐specific ASMR for diseases attributed to diet low in vegetables from 1990 to 2021. (a) ASMR for both sexes. (b) ASMR for females. (c) ASMR for males.
**Figure S3:** Age‐specific temporal patterns of death and DALY rates attributable to diet low in vegetables, 1990–2021. (A) DALY rates attributable to diet low in vegetables by age group from 1990 to 2021. (B) Death rates attributable to diet low in vegetables by age group from 1990 to 2021.
**Figure S4:** Correlation of disease burden attribute to diet low in vegetables and SDI at country level. (a) ASMR of diet low in vegetables by SDI in 2021. (b) ASDR of diet low in vegetables by SDI in 2021.
**Table S1:** The age‐standardized SEV and temporal trends of diet low in vegetables in 1990 and 2021.
**Table S2:** The deaths cases, age‐standardized deaths, and temporal trends of diet low in vegetables in 1990 and 2021.
**Table S3:** The DALYs cases, age‐standardized DALYs, and temporal trends of diet low in vegetables in 1990 and 2021.

## Data Availability

The data from this study can be accessed openly through the GBD 2021 online database (https://vizhub.healthdata.org/gbd‐results/). The code used in this study is available upon reasonable request to the corresponding author.
