# Supplementary material for: The Burden of Disease Attributable to Diet Low in Vegetables From 1990 to 2021: A Global Analysis
Source: Food Sci Nutr. 2026 Sep 15;14(9):e72298. doi: 10.1002/fsn3.72298 (PMC13575977; doi:10.1002/fsn3.72298)
Supplement: Supplementary file 1 [file FSN3-14-e72298-s001.docx]

**The Burden of Disease Attributable to Diet Low in Vegetables from 1990 to 2021: a Global Analysis**

Xindi Wei^1#^, Xiaomeng Zhang^1#^, Xiaolei Lv^1^, Hongchang Lai^1*^, Zhuoli Huang^1*^

^#^Xindi Wei and Xiaomeng Zhang contributed equally as co-first authors.

^1^Department of Oral and Maxillofacial Implantology, Shanghai Ninth People's Hospital, Shanghai Jiao Tong University School of Medicine; College of Stomatology, Shanghai Jiao Tong University; National Center for Stomatology; National Clinical Research Center for Oral Diseases; Shanghai Key Laboratory of Stomatology; Shanghai Research Institute of Stomatology, Shanghai 200011, China

**Supplementary materials**

**Figure S1. The ASR of diet low in vegetables related SEV in countries and territories in 1990 and 2021.** The ASR of diet low in vegetables related SEV in (a) 1990, (b) 2021.

**Figure S2. Joinpoint regression analyses of the sex-specific ASMR for diseases attributed to diet low in vegetables from 1990 to 2021.** (a) ASMR for both sexes. (b) ASMR for females. (c) ASMR for males.

**Figure S3. Age-specific temporal patterns of death and DALY rates attributable to diet low in vegetables, 1990–2021.** (A) DALY rates attributable to diet low in vegetables by age group from 1990 to 2021. (B) Death rates attributable to diet low in vegetables by age group from 1990 to 2021.

**Figure S4. Correlation of disease burden attribute to diet low in vegetables and SDI at country level.** (a) ASMR of diet low in vegetables by SDI in 2021. (b) ASDR of diet low in vegetables by SDI in 2021.

**Table S1. The age-standardized SEV and temporal trends of diet low in vegetables in 1990 and 2021.**

**Table S2. The deaths cases, age-standardized deaths, and temporal trends of diet low in vegetables in 1990 and 2021.**

**Table S3. The DALYs cases, age-standardized DALYs, and temporal trends of diet low in vegetables in 1990 and 2021.**

**Figure S1. The ASR of diet low in vegetables related SEV in countries and territories in 1990 and 2021.** The ASR of diet low in vegetables related SEV in (a) 1990, (b) 2021.

**Figure S2. Joinpoint regression analyses of the sex-specific ASMR for diseases attributed to diet low in vegetables from 1990 to 2021.** (a) ASMR for both sexes. (b) ASMR for females. (c) ASMR for males.

**Figure S3. Age-specific temporal patterns of death and DALY rates attributable to diet low in vegetables, 1990–2021.** (A) DALY rates attributable to diet low in vegetables by age group from 1990 to 2021. (B) Death rates attributable to diet low in vegetables by age group from 1990 to 2021.

**Figure S4. Correlation of disease burden attribute to diet low in vegetables and SDI at country level.** (a) ASMR of diet low in vegetables by SDI in 2021. (b) ASDR of diet low in vegetables by SDI in 2021.

**Table S1. The age-standardized SEV and temporal trends of diet low in vegetables in 1990 and 2021.**

| **Location** | **1990** | **2021** | **1990-2021** |
| --- | --- | --- | --- |
|  | **ASR per 100000 population (95%UI)** | **ASR per 100000 population (95%UI)** | **EAPC (95%CI)** |
| **East Asia** | 25.05 (15.03,31.18) | 3.60 (1.75,6.37) | -7.04 (-7.33,-6.75) |
| **Southeast Asia** | 55.02 (29.71,61.56) | 36.52 (24.18,43.23) | -1.35 (-1.54,-1.17) |
| **Oceania** | 44.63 (21.62,51.13) | 44.33 (22.94,51.10) | 0.10 (0.04,0.160) |
| **Central Asia** | 19.71 (13.34,26.22) | 6.56 (3.89,9.81) | -5.05 (-5.75,-4.35) |
| **Central Europe** | 21.77 (14.26,28.83) | 14.56 (9.15,20.09) | -1.64 (-1.75,-1.53) |
| **Eastern Europe** | 22.60 (15.43,29.55) | 23.28 (15.50,30.37) | 0.35 (0.11,0.59) |
| **High-income Asia Pacific** | 19.06 (11.66,25.81) | 14.59 (8.77,21.18) | -0.52 (-0.74,-0.30) |
| **Australasia** | 30.35 (19.73,37.42) | 30.20 (17.72,38.18) | 0.09 (0.06,0.12) |
| **Western Europe** | 24.36 (16.67,30.82) | 24.92 (16.72,31.61) | 0.16 (0.13,0.19) |
| **Southern Latin America** | 32.23 (20.48,39.12) | 29.30 (18.84,37.01) | -0.23 (-0.29,-0.17) |
| **High-income North America** | 24.35 (15.35,31.70) | 27.16 (16.45,34.26) | 0.72 (0.61,0.83) |
| **Caribbean** | 48.64 (27.80,55.42) | 44.26 (26.13,51.47) | -0.40 (-0.50,-0.31) |
| **Andean Latin America** | 48.23 (25.62,54.59) | 42.02 (25.70,49.29) | -0.57 (-0.64,-0.49) |
| **Central Latin America** | 44.02 (25.96,51.42) | 37.88 (23.74,45.55) | -0.49 (-0.61,-0.38) |
| **Tropical Latin America** | 52.06 (30.00,59.32) | 41.83 (25.9,49.64) | -0.73 (-0.93,-0.53) |
| **North Africa and Middle East** | 21.11 (13.88,26.92) | 16.49 (10.39,21.13) | -1.04 (-1.16,-0.92) |
| **South Asia** | 48.42 (27.44,55.35) | 42.38 (25.12,49.93) | -0.37 (-0.4,-0.34) |
| **Central Sub-Saharan Africa** | 75.67 (35.77,82.36) | 67.47 (33.11,74.18) | -0.53 (-0.61,-0.46) |
| **Eastern Sub-Saharan Africa** | 62.43 (31.54,67.69) | 59.59 (31.94,65.60) | -0.20 (-0.23,-0.17) |
| **Southern Sub-Saharan Africa** | 50.10 (30.30,56.71) | 47.23 (29.5,53.96) | -0.34 (-0.38,-0.29) |
| **Western Sub-Saharan Africa** | 46.36 (27.83,52.68) | 42.17 (26.59,49.23) | -0.29 (-0.34,-0.24) |

ASR, age standardized incidence rate; CI, confidence interval; EAPC, estimated annual percentage change; UI, uncertainty interval.

**Table S2. The deaths cases, age-standardized deaths, and temporal trends of diet low in vegetables in 1990 and 2021.**

| **Location** | **1990** | | **2021** | | **1990-2021** |
| --- | --- | --- | --- | --- | --- |
|  | **Deaths cases no. (95%UI)** | **ASR per 100000 population (95%UI)** | **Deaths cases no. (95%UI)** | **ASR per 100000 population (95%UI)** | **EAPC (95%CI)** |
| **East Asia** | 177030 (97167,255599) | 27.28 (17.10,37.71) | 49787 (21245,94497) | 2.86 (1.26,5.34) | -7.69 (-7.93,-7.44) |
| **Southeast Asia** | 74938 (39841,106485) | 32.02 (18.07,45.1) | 109072 (70095,147390) | 18.44 (11.92,24.76) | -1.83 (-1.95,-1.71) |
| **Oceania** | 975 (448,1404) | 38.58 (19.04,55.06) | 2001 (988,2932) | 29.35 (15.11,42.48) | -0.82 (-0.88,-0.77) |
| **Central Asia** | 6171 (2830,9811) | 14.41 (6.67,22.77) | 3256 (1850,5124) | 5.19 (2.96,8.06) | -4.33 (-5.03,-3.62) |
| **Central Europe** | 17087 (10070,25397) | 12.64 (7.46,18.72) | 15084 (9350,21708) | 6.37 (3.92,9.21) | -2.47 (-2.56,-2.38) |
| **Eastern Europe** | 28505 (13194,44945) | 11.24 (5.15,17.73) | 32061 (16447,49604) | 9.07 (4.65,14) | -0.99 (-1.33,-0.65) |
| **High-income Asia Pacific** | 8322 (4613,12792) | 4.76 (2.73,7.24) | 12332 (6913,19194) | 1.86 (1.02,2.93) | -2.42 (-2.89,-1.94) |
| **Australasia** | 2026 (964,3093) | 8.93 (4.24,13.62) | 2344 (1214,3531) | 3.87 (1.97,5.88) | -2.61 (-2.74,-2.48) |
| **Western Europe** | 48425 (25984,72751) | 8.31 (4.44,12.49) | 63384 (42348,86988) | 5.28 (3.33,7.38) | -1.12 (-1.21,-1.03) |
| **Southern Latin America** | 7892 (5089,10632) | 18.45 (11.97,24.85) | 8840 (6422,11853) | 9.69 (7.03,13.05) | -1.80 (-1.91,-1.68) |
| **High-income North America** | 29126 (15870,44061) | 8.22 (4.49,12.43) | 56478 (37769,77205) | 8.33 (5.59,11.32) | 0.42 (0.32,0.53) |
| **Caribbean** | 6854 (3860,9488) | 28.25 (16.13,38.91) | 11548 (6834,16108) | 21.23 (12.48,29.66) | -0.83 (-0.97,-0.69) |
| **Andean Latin America** | 4130 (2798,5468) | 22.27 (15.51,29.29) | 8162 (5368,11265) | 14.41 (9.49,19.88) | -1.3 (-1.45,-1.14) |
| **Central Latin America** | 13526 (8789,17958) | 18.88 (12.61,25.08) | 30314 (20100,42094) | 12.58 (8.37,17.42) | -1.3 (-1.44,-1.16) |
| **Tropical Latin America** | 21687 (12899,29946) | 26.35 (16.37,35.92) | 30581 (20336,40827) | 12.23 (8.17,16.32) | -2.37 (-2.51,-2.24) |
| **North Africa and Middle East** | 35724 (23411,49295) | 26.01 (17.48,35.59) | 49743 (32983,68993) | 13.14 (8.83,18.42) | -2.39 (-2.52,-2.26) |
| **South Asia** | 118729 (62788,169984) | 23.53 (13.44,33.76) | 235197 (140534,323453) | 17.98 (10.92,24.72) | -0.85 (-0.92,-0.78) |
| **Central Sub-Saharan Africa** | 13215 (6449,19771) | 71.37 (37.96,104.19) | 26840 (13438,39209) | 62.35 (33.63,88.62) | -0.58 (-0.66,-0.5) |
| **Eastern Sub-Saharan Africa** | 40825 (21763,56788) | 64.62 (37.62,88.47) | 58147 (32028,82287) | 42.1 (24.59,58.38) | -1.68 (-1.78,-1.57) |
| **Southern Sub-Saharan Africa** | 7222 (4286,9934) | 29.56 (18.46,40.78) | 16733 (10283,23325) | 33.58 (21.07,45.91) | 0.41 (0.01,0.82) |
| **Western Sub-Saharan Africa** | 23668 (15386,31748) | 31.35 (20.82,41.92) | 39577 (25381,54313) | 23.52 (15.2,32.19) | -1.14 (-1.26,-1.02) |

ASR, age standardized incidence rate; CI, confidence interval; EAPC, estimated annual percentage change; UI, uncertainty interval.

**Table S3. The DALYs cases, age-standardized DALYs, and temporal trends of diet low in vegetables in 1990 and 2021.**

| **Location** | **1990** | | **2021** | | **1990-2021** |
| --- | --- | --- | --- | --- | --- |
|  | **DALYs cases no. (95%UI)** | **ASR per 100000 population (95%UI)** | **DALYs cases no. (95%UI)** | **ASR per 100000 population (95%UI)** | **EAPC (95%CI)** |
| **East Asia** | 4320539 (2244011,6454049) | 536.29 (296.32,768.94) | 837635 (345961,1597481) | 43.78 (18.44,82.3) | -8.66 (-8.99,-8.32) |
| **Southeast Asia** | 2222576 (1073132,3183670) | 802.75 (409.35,1143.77) | 2933475 (1884974,3998881) | 435.48 (281.39,591.88) | -2.03 (-2.18,-1.88) |
| **Oceania** | 30794 (13747,44641) | 948.45 (436.73,1359.62) | 63696 (30458,94112) | 743.7 (363.07,1094.01) | -0.72 (-0.77,-0.66) |
| **Central Asia** | 154294 (72907,245264) | 328.3 (155.57,522.21) | 66324 (36628,107307) | 90.56 (51.09,143.7) | -5.61 (-6.45,-4.77) |
| **Central Europe** | 377448 (217409,568136) | 263.04 (152.62,393.55) | 245992 (146977,361473) | 110.67 (65.7,163.11) | -3.19 (-3.32,-3.06) |
| **Eastern Europe** | 678341 (338064,1050401) | 252.58 (124.67,391.17) | 678454 (350242,1052771) | 200.12 (103.69,308.8) | -1.07 (-1.41,-0.73) |
| **High-income Asia Pacific** | 172767 (88546,272082) | 90.28 (47.69,140.72) | 178655 (96724,279052) | 35.44 (17.31,57.06) | -2.49 (-2.89,-2.09) |
| **Australasia** | 42436 (20956,63865) | 184.17 (90.99,277.25) | 40582 (20407,62317) | 76.02 (37.57,117.04) | -2.77 (-2.93,-2.62) |
| **Western Europe** | 939798 (491789,1419608) | 167.86 (86.89,254.26) | 943337 (579327,1342628) | 93.57 (53.47,133.81) | -1.63 (-1.71,-1.55) |
| **Southern Latin America** | 169722 (105897,230450) | 375.07 (235.12,508.96) | 155567 (106505,212375) | 177.11 (120.1,243) | -2.23 (-2.34,-2.12) |
| **High-income North America** | 622535 (345442,932069) | 184.16 (102.68,275.66) | 1153201 (779749,1570062) | 192.74 (132.8,260.48) | 0.65 (0.53,0.78) |
| **Caribbean** | 175009 (89997,247143) | 667.51 (349.5,941.32) | 286357 (156679,416738) | 536.12 (292.9,781.1) | -0.68 (-0.83,-0.52) |
| **Andean Latin America** | 99767 (60522,136234) | 475.46 (300.79,642.1) | 175622 (109872,250076) | 297.76 (187.78,423.11) | -1.48 (-1.62,-1.33) |
| **Central Latin America** | 333831 (209868,453297) | 396.91 (253.25,535.2) | 701996 (460004,981035) | 278.89 (183.8,389.21) | -1.16 (-1.31,-1.01) |
| **Tropical Latin America** | 603785 (339019,840174) | 626.35 (362.64,866.08) | 723110 (453569,980042) | 280.41 (177.49,379.5) | -2.59 (-2.76,-2.43) |
| **North Africa and Middle East** | 903645 (567336,1248323) | 541.05 (351.15,749.94) | 1244600 (789233,1759517) | 268.49 (172.84,376.46) | -2.51 (-2.64,-2.38) |
| **South Asia** | 3529651 (1790351,5042141) | 574.33 (301.24,820.29) | 6269750 (3586166,8741411) | 416.73 (242.85,580.72) | -1.04 (-1.08,-1) |
| **Central Sub-Saharan Africa** | 387526 (178352,585310) | 1666.09 (812.44,2466.14) | 778566 (355349,1165531) | 1381.31 (670.1,2023.54) | -0.79 (-0.87,-0.7) |
| **Eastern Sub-Saharan Africa** | 1152268 (557929,1642099) | 1489.3 (782.94,2082.88) | 1611778 (810773,2314901) | 915.95 (492.91,1302.05) | -1.92 (-2.04,-1.8) |
| **Southern Sub-Saharan Africa** | 200634 (113789,280076) | 698.2 (400.81,973) | 444560 (260194,630681) | 750.33 (448.38,1051.45) | 0.23 (-0.18,0.63) |
| **Western Sub-Saharan Africa** | 638823 (400663,868393) | 709.44 (455.73,961.24) | 1122434 (674739,1556392) | 531.44 (333.66,732.88) | -1.11 (-1.23,-1) |

ASR, age standardized incidence rate; CI, confidence interval; EAPC, estimated annual percentage change; UI, uncertainty interval.
